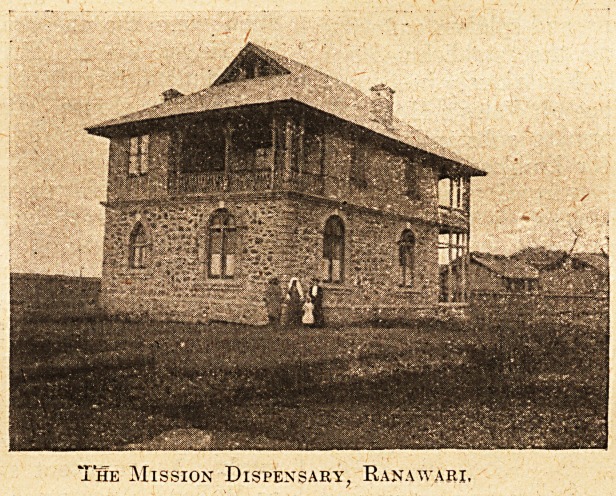# Hospital and Institutional News

**Published:** 1918-08-24

**Authors:** 


### HOSPITAL AND INSTITUTIONAL NEWS.

### KING GEORGE'S FUND FOR SAILORS.

The General Council of King George's Fund for Sailors has allotted the sum of £500 towards Navy House, which is administered in connection with the Royal Naval Barracks, Chatham. Navy House, which has been enlarged and improved to meet the need for increased accommodation, was recently opened by the Duke of Connaught. It is confidently hoped that Navy House in the future will play an even more important part in ministering to the needs of the men of the Fleet stationed at Chatham.

### THE FORTHCOMING HOSPITAL CONFERENCE.

The Council of the British Hospitals Association is circulating a pamphlet on "The Voluntary Hospitals and the Proposed Ministry of Health Bill," which has been prepared by Mr. J. Courtney Buchanan, secretary of the Metropolitan Hospital. The pamphlet is intended not so much to express the considered opinion of the Association as to provide a basis of discussion for the conference to be held on Friday, October 18, at St. Thomas's Hospital, London, at 3 p.m. The committees of constituent hospitals are invited to send any suggestions on practical points and policy to the hon. secretaries of the Association *before September* 30, so that the Council may be able to judge what are the particular matters which the general body of members desires to be discussed at the conference. It is in order to sift opinion, and focus attention on important matters, that Mr. Buchanan's pamphlet has been issued, and the success of the conference will largely depend upon the consideration given to the question between the present time and the day of meeting. Members should think out clearly their own attitude, and formulate in advance their own difficulties, for which reason Mr. Buchanan's ideas should be carefully studied, if only that committees may be definitely able to say where they agree or differ when the time to formulate the Association's policy arrives.

### THE KING AND THE DISABLED.

The first meeting of the board of trustees of the King's Fund for Disabled Officers and Men is to be followed, we understand, by the issue of a general appeal. The Ministry of Pensions is interested, of course, in its success, and it will be remembered that when the King consented to become patron of the Fund he showed his personal interest by the munificent gift of £78,000. A representative body of trustees has been appointed, and the Admiralty, the War Office, and the Air Ministry have been severally invited to nominate a disabled officer and a disabled man. It is, therefore, to be hoped that the interests of the disabled men themselves will be actively represented, and that the work of the trustees will not degenerate into mere officialism, a risk from which such funds are never free. The trustees will co-operate with the Ministry of Pensions, and be responsible for the making of grants, as well as for the general administration.

### BRITISH DOCTORS RECEIVE ITALIAN HONOURS.

At the end of nearly three years' service, the British members of the Royal Italian Military Medical Commission have had conferred upon them by the King of Italy honours as follows: Dr. T. Vincent Dickinson and Dr. James Donelan, Officers of the Order of the Crown of Italy; Dr. Andrew Morton and Dr. Andrew Currie, Cavaliers of the Order of the Crown of Italy. The work of these gentlemen has chiefly been in connection with the examination of the Italian recruits. The Italian authorities take the opportunity of thanking most cordially both the British and the Italian members of the Commission for the care and zeal displayed in this work, and of recording their entire satisfaction with the result.

### AMERICA AND THE ROYAL FREE HOSPITAL.

A grant of £10,000 has been made to the Royal Free Hospital by the American Red Cross Commission for Great Britain to extend the Maternity Department, and it has been decided to take over and extend the Maternity Hospital, hitherto entirely supported by the Duchess of Marlborough, which at present provides for twenty patients. As soon as the necessary arrangements can be carried through it is intended to provide fifty beds for maternity cases. It is also intended to extend at this centre maternity training for the nursing staff of the Royal Free Hospital and other pupils. The board of the Royal Free Hospital keenly appreciates the friendly spirit of the American Red Cross Society in making this grant, which promises an excellent start for an ambitious scheme.

### LOTTERIES AND ITALIAN HOSPITALS.

The defeat of the Lotteries (War Charities) Bill in the House of Commons has led to various suggestions for an alternative and profitable disposal of the Red Cross Necklace. In suggesting that, if the American law allows, a lottery might be held in the United States for which tickets could be purchased, as in the case of the Calcutta Sweep, in this country, Sir Lees Knowles calls attention in the *Times* to the practice of Italy. He states that the Banco Lotto is managed by the Government for the benefit of the hospitals, and defends the practice on the ground that the opportunity thus sanctioned directs an ineradicable human tendency to gamble into harmless channels. The pearl necklace will have to be disposed of, and its fate involves prejudices which voluntary hospitals have been wise in refusing to arouse. The controversy over the necklace, in which they are not directly concerned, is a useful reminder that hospital charity has something more in view than financial success, and that financial success for the hospitals is best secured by eschewing adventitious aids, and relying on public generosity.

### STAFFS, SALARIES, AND THE WAR LOAN.

Among the hospital staffs which have been encouraged to invest in the War Loan are those of the Metropolitan Asylums Board, and it is interesting to record what has been achieved in this direction. A recent list includes twenty-eight names, and every grade in the various staffs appears to be represented. One sister is credited with stock to the value of £55, a staff nurse has £25, a handy-man £5, a scullery maid £4, a probationer £4, a nurse £5, and so on. It is perhaps because the matrons have lately petitioned for an increase of salary that they are not represented on the above list. However, subject to the consent of the Local Government Board, the salaries of matrons are to be increased by £50 a year, and this, when in force, will bring the lowest matron's salary to £150 and the highest to £230. Meantime the shortage of staff remains a pressing difficulty. Applicants are few, and the Board is advised by the Ministry of Labour to spend more on advertising, and to take a more active part in the Women's War Work Exhibitions. A pamphlet for distribution to these exhibitions and to the Employment Exchanges and public libraries is being prepared. Apparently even the increased salaries cannot yet satisfactorily compete with the attractions offered by war work and the pay to be had in Government departments. Women are too apt to forget that war work is temporary, while the Asylums Board institutions continue for ever and ever.

### A WELSH ENTHUSIAST.

Welsh hospitals never seem to lack enthusiasts. Major James German, J.P., a Cardiff coal exporter, although at Barmouth under doctor's orders, is at work on behalf of the Welsh Hospital, Netley. Assisted by Mr. Owen Parry, of Barmouth, he organised a Flag Day, which realised £150, a record sum for the town. Then he held a charity auction, when he sold for £12 pipes which he had previously purchased for 18s., and disposed of a paper necklace made by a little girl visitor for £4. Finally, he spoke, after the Prime Minister's message to the nation had been read, at the local theatre, and his appeal for the institution brought in £18. The result of these enthusiastic efforts is sufficient to endow three beds at Netley Hospital for a year.

### THE VITALITY OF THE VOLUNTARY SYSTEM.

The accounts of any hospital which in these days is able to turn a considerable debt into a balance in hand, without the aid of large legacies, are worth attention even though the success is due perhaps to the capitation grants received for soldiers. The Royal Cornwall Infirmary presents this happy example. After beginning the year with a debt of £484, apart from £544 which was owed to the bank, there was a credit balance on the year's work running well into three figures. Voluntary donations came to the rescue, the Dorrington Convalescent Home at Perranporth, still used as a hostel for munition girls, had led to the investment of an accumulated sum of £300 in War Bonds, collections in churches increased by £200, and £2,559 was received for the treatment of soldiers. The result is a successful financial year which shows the vitality of the voluntary system as much in the varied work performed as in the figures of the balance sheet.

### THE LATEST REGISTER OF CHARITIES.

For the sum of one shilling each copies may be obtained of the revised Supplementary List of Charities, which has been issued by the Charity Commissioners, and covers those entered in the first six months of 1918. This Supplementary List to the Register of Charities may be obtained from the Stationery Office, Imperial House, Kingsway; 37 Peter Street, Manchester; from E. Ponsonby, Limited, 116 Grafton Street, Dublin, and 23 Foster Street, Edinburgh.

### BETTER BREAD FOR WOUNDED SOLDIERS.

When Boards of Guardians whose institutions have been taken over by the military authorities for wounded soldiers keep a watchful eye over the patients' food, defects can be remedied successfully. Some weeks ago the attention of the Southwark Board of Guardians was drawn to the bread supplied to the patients through the Army Service Corps. It was sour and unpalatable, and they at once wrote to the officer commanding, stating that if there was not an alteration in the quality of the bread they would have to supply it from their Newington institution, which they had done at first. A fortnight elapsed. No alteration had taken place, and the Visiting Committee reported that, if anything, the bread was worse. So orders were given for it to be supplied once more from their own bakery. The return to what may be called the "home-made bread" has met with general satisfaction at the hospital. The Guardians are to be commended for their action, for when the Army authorities decided to supply their own bread the Guardians reduced the staff at their bakery, so that the large extra supply has had to be baked by a reduced staff at considerable inconvenience.

### THE PROGRESS OF ARMY DENTISTRY.

The recent statement by Dr. W. H. Dolamore, President of the British Dental Association, that "the Canadian Army is the only army which attempts to send its soldiers to the Front dentally fit," should remind the public of the interesting work which is being carried on at the Ontario Military Hospital, Orpington, Kent, where an elaborate dental clinic has been organised. Apart from the treatment of dental caries and trench mouth, injuries to the jaws are carefully treated, and triumphs of oral surgery are to be seen there. The reconstruction of a mouth is perhaps the most delicate problem of oral surgery, since the mouth has functions to perform as well as appearances to present. The mobility required in the jaws themselves and the relation of the mouth to the organs of speech complicate the surgeon's work, and it is gratifying to remember that the success gained has come to the nation which has been the pioneer in army dentistry.

Canada has much to be proud of in the thoroughness of her dentistry, as well as in the ingenuity and skill of her oral surgeons. The dental mechanics at Orpington have the rank of non-commissioned officers, and some account of their work was given in a recent number of *Canada*.

### A CRY FROM KASHMIR.

We have received from Miss E. M. Newman, lady-in-charge of the C.E.Z. Mission Dispensary, Ranawari, Srinagar, Kashmir, India, the following account of a fire which in April last destroyed the hospital and dispensary. It appears not to be a case of incendiarism, designed by the thieves who broke into the store-room, but caused by the upsetting of a tin of kerosene oil which was found among the wreckage. "We were working in the hospital," says Miss Newman, "till the bell rang for dinner, when we went over to the House for our meal. An alarm of fire quickly followed, and we rushed out to see, never thinking it was our own place. The whole of the middle floor of the hospital was in flames. After a careful investigation the superintendent of police came to the conclusion that the fire might have been an accident caused by some person or persons who came to steal and broke open the store-room." The buildings, we learn, were built by voluntary contributions, and have now been burnt "to the ground." Miss Newman begs for support from those at Home, and asks us to say that "the very smallest sums will be thankfully received to help in our trouble and loss. All money will be acknowledged by return of post and in our annual report." It should be sent direct to the Secretary of the Church of England Zenana Missionary Society, 27 Chancery Lane, stating that it is for the rebuilding of the Ranawari Mission. It is a far cry from Kashmir. But it has reached us, and we hope it will touch our readers.

The Mission Dispensary, Ranawari.

### A PERMANENT STOP-GAP.

It is now thirty years since Mr. E. S. Johnson became honorary secretary of the Derbyshire Convalescent Home at Matlock. It is interesting to recall that Mr. Johnson was originally a stop-gap, and took up the work at first temporarily. However, he soon made it his own, and still takes as much pleasure in the work as he did a generation ago.

### WELFARE WORK IN ITS INFANCY.

In his inaugural address to the Welfare Conference at Oxford, Dr. E. L. Collis, Director of the Welfare and Health Section of the Ministry of Munitions, discoursed on the ethics of industrial welfare. Industry, not conquest, he said, was the means by which man had controlled natural forces to his own advantage, and the delegation of industry to a slave class had led to the downfall of the great empires. The truth was that industry was necessary to foster the intelligence of the race, and its true value lay not in the wealth produced, but in the opportunity for healthy activity which industry afforded. Industry must be freed from the shackles of the economist's idea of it, and the welfare movement was the means whereby this freedom might be won. Welfare work regarded the physical and the intellectual life of the worker, with his recreation as with his housing or his food, but it was still in its infancy. A sketch of what further aims it should set before it would be welcome. For, like the out-patient department and the hospitals' Samaritan Funds, it will develop in many directions.

### A BRIGHTON HOSPITAL'S JUBILEE.

The Royal Alexandra Hospital for Sick Children at Brighton has just celebrated its thirty-seventh anniversary in its fine existing premises in Dyke Road, but the work has been carried on since 1868, and thus this year the hospital celebrates its jubilee. The occasion is to be marked by an endeavour to raise at least £5,000 to enable it to "carry on." The president, the Earl of Chichester, is making the appeal, to which not only Brighton and Hove, but the county of Sussex generally, ought to respond.

### THE "AMERICAN RED CROSS BULLETIN."

Institutional workers should be interested in the *American Red Cross Bulletin*, a miniature weekly journal of eight pages now published in London. The aim of its illustrated contents is to acquaint workers in one department or district with what is happening in the others, but there is a larger interest for English readers. The American point of view is made apparent in the American accounts of work in this country. In describing the new American military hospital at Sarisbury Court, near Southampton, the work was said to be "of what Englishmen call modern construction," because it is only thirty-five years old. American Red Cross work in France and in Italy is also touched on, and the visit of the Japanese Red Cross Mission, which has arrived in this country from America, is sympathetically dealt with.

### THE RELIEF OF WAR DISTRESS.

In their report of the administration of the National Relief Fund during the half-year ending March 31 last, the Executive Committee do well to emphasise a note of warning in regard to the outlook for organised relief of distress caused by the war. There is a danger of its being assumed that the excellent conditions of employment which have prevailed during the last three years are permanent, and of support for distress funds being consequently unnecessary. The committee realise this, and endeavour to correct the mistake by declaring that already indications have appeared of the rapid approach of a state of things which will inevitably greatly extend relief operations; indeed, they foresee that in the near future the demands upon their Fund for the relief of civil distress may prove far more serious than at any period since the beginning of the war. So far the tendency towards neglect of this kind of public assistance has not been unduly manifested in the case of the National Fund, though there was a fall in the subscriptions received during the half-year to £35,289, compared with £47,464 for the previous half. The aggregate receipts of the Fund up to March 31 last were £6,348,577. The total issues up to the same date for naval and military relief amounted to £2,999,899, and for civil relief to £707,174. Considerable sums were devoted in the course of the half-year to the assistance of hospitals, these grants including £16,000 for the purchase of the freehold site of the Queen's Auxiliary Hospital for sailors and soldiers suffering from facial injuries, towards the establishment of which institution £10,000 had previously been contributed; £5,000 to the Prince of Wales's Hospital, Cardiff, an institution performing substantially the same functions in Wales for limbless sailors and soldiers as Roehampton is performing in England and Erskine House in Scotland; £5,000 to the Lingfield Epileptic Colony; and £1,000 to an Edinburgh branch of St. Dunstan's Hostel.

### CURIOUS AIR-RAID EFFECTS.

The record of the Committee's operations in the field of civil distress is not the least interesting part of the report. The amounts issued to local committees under this head were £13,115 in London; £12,595 in English counties; £800 in Welsh counties; and £30,716 in boroughs and districts in England and Wales. The size of the sums granted to East Coast districts—for example, £3,000 to Durham, £3,000 to Kent, £1,700 to Norfolk, £7,700 to Great Yarmouth, £2,250 to Lowestoft, £7,300 to Margate, and £3,800 to Ramsgate—tells its tale of the effects of enemy visitations especially on seaside resorts. This summer has seen a return to a more normal state of things in these places, but the lodging-house keepers and others largely dependent upon season visitors are still in sore need. Special assistance to these for the payment of rent and mortgage interest has hitherto been accorded out of a fund generously provided by the Canadian Government, but that fund is now exhausted and the expenditure under the scheme will in future fall upon the National Relief Fund. The report contains a further and rather unexpected reminder of air raids in the statement that children in certain quarters of London and the outlying districts had in many cases because of bomb-dropping suffered in health, mainly as a result of exposure or from nervous strain and loss of sleep. Happily there were comparatively few of the patients whose cure could not be assured by a country holiday, and the Committee made a grant of £5,000 to the Children's Country Holiday Fund for their benefit, while assistance was given to the Invalid Children's Aid Association and the St. Nicholas Home at Chailey to deal with the more serious cases. The total special grants made to Local Representative Committees for the alleviation of distress caused by enemy air raids was £15,620.

### WAR BONUS TO WORCESTER NURSES.

To avoid misconception in respect to the war bonus of £3 a year granted by the committee of the Worcester General Infirmary to the nursing staff, as recorded in The Hospital of August 3, it should be explained that until July no war bonus had been given. This led to the application being made, which, we were glad to note, was granted by the committee.

### WILLESDEN'S MUNICIPAL CLINIC.

"I came here this afternoon because you have the most perfect, comprehensive, and complete scheme of maternity and child-welfare that has, as yet, been developed by any Urban District Council." Such were the encouraging words spoken by Mr. W. Hayes Fisher, M.P. (President of the Local Government Board), in opening a municipal clinic at Acworth House, High Road, Willesden Green, last week. The district, he added, had attained that perfection in the care of expectant and nursing mothers and children which was being enjoyed by some of our largest towns. He was, he contended, spending his time in a most official manner by visiting Willesden and paying his tribute to the model way in which it was meeting the demand for making the child-life of this country its greatest national asset. To encourage, as he was wont to do, county by county, town by town, and urban district council by urban district council, brought about a healthy rivalry in that most interesting work. A competent staff of health visitors, such as Willesden possessed, was the very foundation of the work of maternity and child-welfare.

### THIS WEEK'S DRUG MARKET.

There are few price changes of importance to note this week, and transactions are limited to a small volume. Jamaica sarsparilla of the first quality is rather dearer. The demand for quinine is greater but the price is maintained. Menthol is tending upwards in price and there is more activity in this market. Makers of bismuth preparations report that the demand is normal; potassium and sodium iodides continue to be in good demand. There is no change in the market position of sugar of milk at present, but further developments are expected.

### TO OUR READERS.

Contributions are specially invited from any of our readers to these columns. They should deal with topical subjects and news. They must be authenticated for the information of the Editor only. The minimum payment if published is 5s. There is no hard-and-fast rule as to space, but notes of about twenty lines in length are preferred.

## Figures and Tables

**Figure f1:**